# Antibacterial efficacy of local plants and their contribution to public health in rural Ethiopia

**DOI:** 10.1186/s13756-017-0236-6

**Published:** 2017-07-28

**Authors:** Gutema Taressa Tura, Wondwossen Birke Eshete, Gudina Terefe Tucho

**Affiliations:** Department of Environmental health Sciences and Technology, Jimma University, Jimma, Ethiopia

**Keywords:** Plants, Phytochemical, Hand hygiene, Antibacterial, Antimicrobial resistance, Public health

## Abstract

**Background:**

Proper hand hygiene with soap and detergents prevents the transmission of many infectious diseases. However, commercial detergents are less likely to be accessible or affordable to poor people in remote rural areas. These people traditionally use some plant parts as a detergent even though their antibacterial activity has not been yet investigated. Therefore, this study aims to determine the antibacterial activities of some of the plants against bacteria isolated from humans.

**Methods:**

Plants selected for this study are *Phytolacca dodecandra fruits*, *Rumex nepalensis* leaves, *Grewia ferruginea* bark and leaves. The samples of these plants were collected from rural areas of Jimma town based on their ethno-botanical survey and information on their local use. Acetone was used as a solvent to extract the bioactive constituents of the plants. The antibacterial activities of the plants were evaluated against reference strains and bacteria isolated from humans using disc diffusion and macro dilution methods.

**Results:**

The plant extracts have shown varying antimicrobial activities against the bacterial species tested. Susceptibility testing shows zones of inhibition ranging from 8.0 ± 1.0 mm to 20.7 ± 5.5 mm. The MIC and MBC of the plants against the bacterial species tested were 3.13 and 12.5 mg/ml respectively. These variations are attributed to different concentrations of the bioactive constituents of the extracts like saponins, tannins, flavonoids and terpenoids.

**Conclusion:**

The studied plants can contribute to achieve better personal hygiene since they are effective against different bacterial agents and are freely available in rural areas.

## Background

Soap has been used for personal hygiene for many centuries. The effectiveness of soap to clean dirt is based on its detergent properties. However, soaps containing antiseptic agents in addition to detergents are available since the 19th century [1, 2]. In particular, hands perform several activities through which they come into contact with contaminated objects. Hands will be routes for disease transmission if proper hand hygiene is not performed. In fact many infectious diseases are easily transmitted through hand contacts from the immediate environment [3, 4]. Many of the diseases in developing countries are related to fecal-oral transmission attributed to insufficient personal hygiene [5]. Hand hygiene is the simplest, most cost effective and easily applicable measure that can reduce the risk of spread of infectious diseases [6–8]. Nevertheless, the effectiveness of hand hygiene depends on the habit of using soap during washing [7, 9]. However, better microbial removal and hygiene might be more achieved when antimicrobial detergents are used instead of plain soap and water [1, 7].

There are many effective antimicrobial cleaning products available to provide better cleaning services. In particular, alcohol based cleaning agents are effective against gram negative and gram positive bacteria despite some limitations related to short time residual effects [10, 11]. Nevertheless, most effective commercial antimicrobial agents are less likely accessible or affordable to poor people living in remote rural areas of developing countries to meet the goal of hand hygiene [12]. Alternatively, different plants with antimicrobial properties can replace expensive commercial antimicrobial products [13, 14]. Most plant products are effective including against harmful resistant micro-organisms [15].

People in rural areas have a rich tradition of using different parts of plants for personal hygiene. For instance, *Phytolacca dodecandra (P. dodecandra)* fruits, *Rumex nepalensis (R. nepalensis)* leaves and *Grewia ferruginea (G. ferruginea*) leaves and bark are some of the most commonly used plants in different rural areas of Ethiopia for cleaning purposes (i.e. bathing, washing clothes etc.). There are a lot of effective different plants used for personal hygiene in different parts of the world [16–19]. Several medicinal plants have been studied for their antibacterial activities against different pathogenic bacteria species [20–22]. Many of them were also found to be effective against resistant microbial strains [16, 23]. However, the antibacterial activities of plants used for personal hygiene have not been yet determined. These plants can be cost effective alternatives to modern detergents if their antibacterial activities are determined and promoted. Studying their antibacterial activity is not only to promote them as alternatives but also to preserve indigenous knowledge about the use of local plants for personal hygiene [24]. Therefore, this study aims to evaluate the antibacterial activities of some plants used for personal hygiene against bacterial colonizing the skin. The results can be vital in the promotion of low cost and effective cleaning materials in rural areas and in preserving indigenous knowledge about plants used for personal hygiene.

## Methods

### Plant sample collection and preparation

Different parts of healthy test plants of *P. dodecandra* (local name “Andoode”) fruits, *R. nepalensis* (local name “Timiji”) leaves, *G. ferruginea* (local name “Dhoqonu”) leaves and bark were collected from rural areas within the Jimma zone. The selection of the test plants was based on ethno-botanical surveys and relevant information of traditional use of the plants as detergents [21, 25]. The parts of the plants considered for sampling are frequently used for cleaning purposes.

The collected samples were washed under running clean tap water to eliminate adhering dust and any foreign particles and shaded to dry at room temperature for about 7–14 days. The dried samples were grinded with a mechanical grinder, sieved with a 2.5 mm sieve size and stored at 4 °C until considered for extraction [26]. The bioactive constituents of the plants were extracted with acetone. Acetone was selected as a solvent based on its low toxicity, easiness of extraction and easy evaporation [27]. The extraction was performed by dissolving 200 g of each plant’s powder in 700 ml of acetone, shaken at constant speed of 300 rpm (HY-5A Maneuver style vibrator shaker) and filtered with Whatman No.1 filter paper. The contents were then dried and weighed for their extract yields and stored at 4 °C for microbial assay. The percentage extract yields of the plants were calculated as:$\mathrm{Percentage}\ \mathrm{extract}\ \mathrm{yield}\ \left(\%\right)=\frac{\mathrm{Weight}\ \mathrm{of}\ \mathrm{dried}\ \mathrm{extract}\ }{\mathrm{Weight}\ \mathrm{of}\ \mathrm{dried}\ \mathrm{powder}}\ \mathrm{X}100$ (Table 1).

**Table 1:** Ethno-botanical and relevant information of the plants

Name			Parts used	Traditional use	Picture of the plant part
Scientific	Family	Local			
*Phytolacca dodecandra*	Phytolaccaceae	Andoodee (Indodi)	Fruit	Used for washing of clothes, hands and body	
*Rumex nepalensis*	Polygonaceae	Timijii (Tult)	Leaf	Used for washing of hands and hair	
*Grewia ferruginea*	Tiliaceae	Dhoqonu (Lenkoata)	Leaf	Used for washing hair	
*Grewia ferruginea*	Tiliaceae	Dhoqonu (Lenkoata)	Bark	Used for washing hair	

### Phytochemical screening

The phytochemical screenings of the plants were made based on qualitative methods used in other studies [28–30]. Saponins, tannins, flavonoids and terpenoids were bioactive compounds considered for identification based on their antimicrobial activities [31–35]. The concentration of the constituents was determined based on their relative color strength; the deeper the color the stronger the concentration of the constituents in the extracts.

Identification of Saponins was made by using a foam test by adding 5 ml of distilled water to 0.5 g of the extracts, shaken vigorously and observed for its frothing. Again three drops of olive oil were added and shaken vigorously for the formation of emulsions indicating the presence of saponins. The test for identification of tannins was done by mixing 0.5 g of the extracts with distilled water and heating on a water bath until the extracts were fully dissolved. The formation of a dark green color indicates the presence of tannins upon addition of 0.1% ferric chloride. Identification of the presence of flavonoids was made by adding 0.2 g of the extracts to 2 ml of 2% solution of NaOH. An intense yellow color was formed which turned colorless upon addition of few drops of diluted HCl indicating the presence of flavonoids. Finally, identification of terpenoids was made by mixing 0.2 g of the extracts with 2 ml of chloroform (CHCl_3_) and 3 ml of concentrated sulfuric acid (H_2_SO_4_) whereby the formation of a layer with a reddish-brown color interface indicates the presence of terpenoids.

### Isolation and identification of bacteria species

Isolation and susceptibility testing of the bacterial species were done by using standardized procedures [36–38]. Isolation of the test bacteria was done by using sterile cotton swabs soaked in 0.85% sterile saline solution. The samples were taken from palms, fingers and fingernails of the left and right hands of individuals working in hospital and food handling activities and preserved at 4 °C in saline solution.

Prior to culturing, MacConkey agar, Mannitol salt agar, Xylose Lysine Desoxycholate agar (XLD agar) and selenite F broth were prepared according to the instruction of the manufacturer and the operating standard procedures [38, 39]. Subsequently, the swab samples were soaked in saline solution, vigorously shaken and 0.2 ml of the solution was cultured on the media and incubated in an inverted position at 37 °C for 24 h. The media containing inoculum was then observed for the formation of distinct isolated colonies for further sub-culturing. Colonies for sub-culture were considered based on the colony morphology of culture positive samples. Accordingly, Lactose fermenting colonies (LFCs) and non-lactose fermenting colonies (NLFCs) were characterized by pink color and pale color respectively on MacConkey agar. Small colonies surrounded by yellow zones or colonies changed the color of Mannitol Salt Agar (MSA) to yellow and white creamy colonies were also identified. Following gram staining, different biochemical and enzymatic reaction tests were done to identify bacteria into species. Therefore, we used oxidase, catalase, Klinger iron agar (KIA), lysine iron agar (LIA), Indole, Motility, Citrate, Urease, Triple sugar iron Agar (TSIA) and TSIA tests.

Due to resource constraints, classification of bacteria was done using the following basic biochemical approach [4, 38, 40]. Accordingly, the identification of *Salmonella* species was made by culturing the colony on Xylose Lysine Desoxycholate agar media enriched with a Selenite broth; thus colonies with a black center were identified as *Salmonella* species. Identification of *E.coli* was made by using MacConkey salt agar and fermentation of lactose with the formation of flat dry pink of irregular colonies. Identification of *S.aureus* was made by using Mannitol salt agar with the subsequent formation of yellow/golden colored colonies. *P. aeruginosa* identification was made by using nutrient agar and MacConkey agar. Colonies with large and irregular opaque bluish-green pigment on nutrient agar and non-lactose fermentation with colorless colonies on MacConkey agar were identified as *P. aeruginosa*. These species of bacteria are responsible for many communicable diseases in developing countries. In addition, most of them are resistant to ordinary antibacterial agents and are considered for the current antibacterial activity testing.

*E. coli (ATCC 25922), P. aeruginosa (DSMZ 1117), S. typhimurium (ATCC 13311) and S. aureus (ATCC 25923)*, all American Type Culture Collections were obtained from the Microbiology lab of Jimma University as reference bacteria strains for a quality control.

### Antibacterial activity testing

Inoculums for antibacterial testing were prepared by transferring pure bacteria strains grown on nutrient agar media to 5 ml sterile physiological saline solution (0.85% NaCl *w*/*v*). The suspended turbidity was adjusted to 0.5 McFarland standards corresponding to 1.5 × 10^8^ CFU/ml [41]. The antibacterial activity testing of the plants was done by using a disc diffusion method [37]. The standardized suspension of bacterial strains of 1.5 × 10^8^ CFU/ml was prepared and diffused on the Mueller Hinton agar (MHA) media with sterile swabs. Sterile filter paper discs of 6 mm diameter were impregnated with a 200 mg concentration of the plant extracts dissolved in 1 ml of DMSO, then placed on swabbed agar and incubated at 37^o^ C for 24 h. The diameters of zones of inhibition were measured in millimeters using a ruler and the average of triplicate results were presented. A positive and negative control was done by 1% phenol solution and DMSO (without plant extracts) respectively.

### Determining MIC and MBC of the plants

The Minimum Inhibitory Concentration (MIC) and Minimum Bactericidal Concentration (MBC) of the plants were determined based on standard procedures and cited literatures [42–44]. Accordingly, a stock solution with the concentration of the plant extracts of 200 mg in 1 ml of DMSO was diluted using a two-fold serial dilution. This provides the series of test concentrations of 100, 50, 25, 12.5, 6.25 and 3.13 mg/ml respectively. The plant’s antibacterial activity testing was done by incubating each concentration of the extract with inoculum containing 0.1 ml of microbial cell at 37 °C for 24 h [45, 46]. The smallest concentration that inhibited the growth of the test bacteria was considered as MIC of the plants. The MBC of the plants was determined by transferring inoculums from MIC tubes to freshly prepared nutrient agar, incubated for 24 h at 37 °C with different concentrations of the extracts [23]. The smallest concentration of the extracts with no visible bacterial growth after incubation was taken as MBC.

## Results

### The extract yields of the plants and their phytochemical constituents

The method used for the extraction achieved varying extract yields ranging from 6.40 g to 15.47 g (Table 2). The highest percent extract yield was obtained from *P. dodecandra* fruits (7.74%) and the lowest (3.2%) was from *G. ferruginea* bark. Variations were also observed in the concentration of the phytochemical constituents of the plant parts and species. Phytochemical constituents are bioactive compounds with many antibacterial activities. Accordingly, all the evaluated plant species contain saponins, tannins and flavonoids in varying concentrations. However, terpenoids were not found in *P. dodecandra* fruits and *R. nepalensis* leaves (Table 3).

**Table 2:** The extract yields of the plants

S.No.	Plant parts	Weight of powder (gram)	Weight of extract (gram)	Percent extract yield (%)
1	*P.dodecandra fruit*	200	15.47	7.74
2	*R.nepalensis leaves*	200	7.10	3.55
3	*G.ferruginea bark*	200	6.40	3.20
4	*G.ferruginea leaves*	200	11.14	5.57

**Table 3:** Phythochemical constituents of the plants

S. No.	Phytochemical components	*P.dodecandra* fruit	*R.nepalensis* leaf	*G.ferruginea* bark	*G.ferruginea* leaf
1	Saponins	++	+	+++	+++
2	Tannins	++	+++	++	+
3	Flavonoids	+++	+	+++	++
4	Terpenoids	−	−	++	+++

Key: = absent; + = present in small amount; ++ = Present in moderate amount; +++ = present in high amount

### Antibacterial activities of the plants

**Fig. 1:**
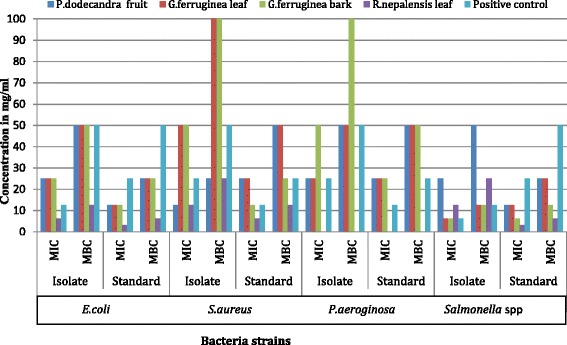
MIC and MBC of the test plants and the positive control against different bacterial species

The antibacterial activities of the plants are shown in Table 4. The data shows huge antibacterial activity variation among different plant species and parts. The extracts of the plants achieved varying zones of inhibition against both isolate and reference (standard) bacteria species. The highest zone of inhibition (20.7 ± 5.5 mm) was achieved with *R. nepalensis* leaves extract against reference *Salmonella* strain (*S. typhimerium*). The zones of inhibition *of P. dodecandra* fruits, and *G. ferruginea* leaves and bark against different bacterial species range from 9.0 ± 1.0 to 16.7 ± 1.2 mm (Mean ± SD). However, all of them achieved a relatively smaller zone of inhibition against isolated bacteria than their reference counterparts. The MIC and the MBC of the studied plants are shown in Fig.1. Their MIC ranges from 3.13 mg/ml to 50 mg/ml while their MBC are between 6.25 mg/ml and 100 mg/ml. This implies that a higher concentration is needed for killing bacteria than for inhibiting their growth. The *G. ferruginea* leaves and bark extract showed the lowest MIC and MBC against *Salmonella* species while *R. nepalensis* similarly showed the lowest values against *E. coli*. Relatively, larger MIC and MBC were obtained with the extract of *G. ferruginea* and *R. nepalensis* against *S. aureus* and *P. aeruginosa.*

**Table 4:** Zone of inhibition (mean ± SD, *n* = 3) of the plants extracts in mm

Extracts	Bacteria strains							
	*E.coli*		*S. aureus*		*P. aeruginosa*		*Salmonella* spp.	
	Isolate	Ref.	Isolate	Ref.	Isolate	Ref.	Isolate	Ref.
*P.dodecandra* fruits	9.7 ± 0.6	11.3 ± 1.5	10.3 ± 2.1	12.3 ± 2.3	9.3 ± 2.5	12.0 ± 1.0	11.0 ± 1.0	16.3 ± 0.6
*G. ferruginea* leaf	10.0 ± 1.0	12 ± 0.0	9.7 ± 0.6	11.7 ± 0.6	9.0 ± 1.0	10.7 ± 1.5	11.0 ± 1.0	14.3 ± 0.6
*G. ferruginea* bark	8.0 ± 1.0	12.0 ± 2.6	9.0 ± 1.0	11.3 ± 2.9	9.7 ± 2.5	10.3 ± 1.2	9.3 ± 0.6	16.7 ± 1.2
*R. nepalensis* leaf	9.0 ± 1.0	10.3 ± 0.6	9.0 ± 1.0	12.0 ± 3.5	NI	NI	10.0 ± 1.0	20.7 ± 5.5
Phenol	7.3 ± 0.6	8.7 ± 1.5	8.0 ± 1.0	8.7 ± 1.2	7.7 ± 0.6	8.0 ± 1.7	5.0 ± 4.4	5.0 ± 4.4
DMSO	0	0	0	0	0	0	0	0

Key: ± SD = Standard Deviation, Ref. = Reference bacteria strain, NI = No Zone of inhibition, n = number of replicates

## Discussion

### Evaluation of the antibacterial activities of the plants

Among modern methods of extraction, maceration is effective in extracting bioactive compounds at room temperature [47]. These compounds contain broad spectrum antibacterial agents acting against different species of bacteria [34, 35, 48, 49]. Different plant species contain different concentrations of bioactive compounds (Table 3). The variation in antibacterial activity against both test and reference bacteria is found to be attributable to variation in bioactive constituents of the plants. The variation was also significant even within the same plant when evaluated against different bacteria species. The antibacterial activity of *R. nepalensis* against *Salmonella* species (*S. typhimurium*) and *P. aeruginosa* is a good example. This plant showed the highest inhibition against *Salmonella* species and no inhibition against *P. aeruginosa.* The variation can be attributed to the high concentration of tannins and the lack of terpenoids in this plant. Nevertheless, most plant extracts achieved smaller zones of inhibition against test bacteria than reference bacterial species due to probability of antibacterial resistance with isolate species. However, all the evaluated plant extracts possess antimicrobial activity with high susceptibility pattern of low MIC and MBC. Their low MIC and MBC indicates their high efficacy against nonresistant and resistant bacteria species [50]. In particular, plants with a high concentration of flavonoids (i.e. *P. dodecandra and G. ferruginea*) have shown better antibacterial activity against isolate bacterial species than the remaining test plant species and parts. Literature reported flavonoids as bioactive compounds with high antimicrobial activities against resistant strains [51, 52].

The bioactive constituents have different mechanism of action against bacterial cells. Tannins and Flavonoids act on bacterial cells through the formation of a complex with cell walls, binding to proteins, disruption of membranes and inhibition of enzymes. Antibacterial effects of saponins are achieved through inactivation of extracellular medium and membranes of the bacterial cell [13]. Saponins-rich extracts are less active against *S. aureus* compared to gram negative bacteria like *E.coli* and *P. aeruginosa* [53]. Studies indicated that most pathogenic bacteria such as *S.aureus* and *E.coli* isolated from the hands of health workers are resistant to many antimicrobial agents [54, 55]. However, the plants evaluated in the current study showed moderate to highest antibacterial activities against these organisms probably due to availability of bioactive compounds in high concentration. In particular, plants containing tannins, flavonoids and saponins are effective against resistant bacterial species [16]. This shows the effectiveness of the studied plants against various bacterial species.

### Contribution of the plants to rural sanitation and public health

Most people living in rural areas of developing countries do not have sufficient income to afford the costs of modern antibacterial detergents. Moreover, most of them do not have sufficient awareness of the use of antibacterial detergents as a first line of defense against many communicable diseases [9, 56]. In addition, most of them do not have access to basic sanitation and clean water supply. Lack of access to basic sanitation and clean water can be solved by building the system providing the services. However, fecal-oral contamination and transmission of related diseases are inevitable if good hand hygiene is not practiced [57]. In areas where commercial detergents are not available or affordable the studied plants can be used as an alternative. These plants are available in most rural areas throughout the year to use without any costs. Moreover, research shows that *S. aureus*, *E. coli* and *P. aeruginosa* species are resistant to most antibacterial agents [27]. However, all the test plants in the current study have shown antibacterial activity against these organisms with varying concentrations. This implies the possibility to use the ingredients of the plants in the formulation of commercial antibacterial detergents. However, the current study was not designed to provide its efficacy against resistant bacteria in a short contact time. Therefore, further investigation is needed to determine their efficacy with a short contact time. Nevertheless, the results are robust in promoting plant materials for hand hygiene in remote rural areas where commercial detergents are not available or not affordable to the poor.

## Conclusion

The current study evaluated the antibacterial activities of three plant species traditionally used as detergents in rural areas of Ethiopia. All the test plant species have shown moderate to high antibacterial activity against the test bacteria species. *P.dodecandra* fruit and *G.ferruginea* bark and leaves have shown zones of inhibition ranging from 8 to 11 mm against *E. coli, S. aureus, P. aeruginosa* and *Salmonella* species. *R. nepalensis* also has shown zones of inhibition ranging from 9 to 12 mm against all test bacterial species except *P. aeruginosa.* In addition, all of them achieved better antibacterial activity with the smallest concentration of the extracts (i.e. lowest MIC and MBC). The difference in antibacterial activity of the plants can be attributed to varying availability of the bioactive constituents. However, all of them contain effective bioactive constituents such as tannins, flavonoids and saponins in varying concentration. These constituents are effective against different bacterial species including resistant strains. Therefore, all the test plants are strong enough to replace commercial detergents and achieve good personal hygiene in rural areas where the accessibility or affordability of commercial detergents are limited or absent. These plants are abundantly available in rural areas to be a promising source of commercial antimicrobial agent production if further investigation is considered.

## Abbreviations

DMSO: Dimethylsulfoxide
KIA: Klinger iron agar
LFCs: Lactose Fermenting Colonies
LIA: Lysine iron agar
MBC: Minimum Bactericidal Concentration
MHA: Mueller Hinton Agar
MIC: Minimum Inhibitory Concentration
NLFC: Non-lactose fermenting colonies
XLD: Xylose Lysine Desoxycholate
